# Perceived prevalence of bullying as a proxy for normalized disrespect: anti-values, social cleavages, and the normative climate of Kazakhstani society evidence from a nationwide survey

**DOI:** 10.3389/fsoc.2026.1872912

**Published:** 2026-08-25

**Authors:** Zhanna Khamzina, Yermek Buribayev

**Affiliations:** Department of Law, Zhetysu University Named After I. Zhansugurov, Taldykorgan, Kazakhstan

**Keywords:** anti-values, bullying, ethical values, Kazakhstan, moral economy of disrespect, normative climate, perceived prevalence, social inequality

## Abstract

**Introduction:**

Research on bullying has focused primarily on schools and workplaces. This cross-sectional study analyzes how adults in Kazakhstan assess bullying prevalence and how their assessments relate to the perceived prevalence of nine other socially disapproved practices, perceived value differences between social groups, and endorsement of four ethical values.

**Methods:**

Data came from a nationwide, quota-controlled computer-assisted personal interviewing survey conducted from May to June 2025 (*N* = 3,123). Descriptive analysis retained four response categories. Binary logistic regression coded mass/ubiquitous and frequent responses as *Y* = 1 and isolated manifestations as *Y* = 0; exclusion of 325 difficult-to-answer responses produced an analytical sample of 2,798. Predictors included the anti-value prevalence cluster, perceived value differences between officials and the public and between affluent and low-income groups, an ethical-core indicator, sociodemographic covariates, and regional fixed effects. Exact cross-classification examined the coexistence of the ethical-core criterion and the four-category bullying response.

**Results:**

In the full sample, 69.3% classified bullying as mass/frequent, 20.3% as isolated, and 10.4% selected “difficult to answer.” Relative to the low anti-value cluster, adjusted odds ratios were 12.75 (95% CI 8.36–19.44) in the medium cluster and 64.07 (95% CI 42.50–96.58) in the high cluster. Large perceived value differences between affluent and low-income groups were associated with higher odds of a mass/frequent classification (aOR = 2.18, 95% CI 1.16–4.09); estimates for value differences between officials and the public were nonsignificant. Cross-classification identified 1,589 respondents who endorsed all four ethical values as very important and classified bullying as mass/frequent, representing 50.9% of the full sample and 69.1% of respondents meeting the ethical-core criterion.

**Discussion:**

The survey records a concentrated pattern of mass/frequent assessments across bullying and other anti-values, along with an independent association for perceived affluent-poor value differences.

## 1 Introduction

Bullying research has examined victimization, aggression, bystander behavior, and institutional conditions in schools and workplaces. It defines bullying as harmful conduct involving identifiable participants and informs psychological, educational, and organizational interventions.

Societal assessments of bullying prevalence address a different level of analysis. Adults who classify bullying as routine or widespread report a social environment in which humiliation, exclusion, and abuse of power appear recurrent across everyday settings. This study examines that perceived societal environment through the concept of normative climate.

Behavioral bullying involves repeated or systematic aggression, humiliation, exclusion, or another abuse of power within an asymmetric relationship (Olweus, 1993; Menesini and Salmivalli, 2017; Andrews et al., 2023). The survey measured adults' assessments of the prevalence of the everyday category “bullying” in Kazakhstan. The item records perceived typicality of disrespect at the societal level; it does not measure personal victimization, observed incidence, or moral approval.

International research treats bullying as an educational and public health concern because repeated victimization causes psychosocial and educational harm, and socioeconomic and institutional conditions affect its distribution (Menesini and Salmivalli, 2017; Hosozawa et al., 2021). Research in Kazakhstan and other post-Soviet settings has concentrated on school-age populations, including a TIMSS (Trends in International Mathematics and Science Study)-based analysis of eighth-grade victimization (Kaliyeva, 2026). Evidence on adult perceptions of bullying prevalence at the societal level remains limited.

The analysis reports the four-category distribution of bullying assessments. Logistic regression estimates associations between a mass/frequent classification, the perceived prevalence of nine other anti-values, and perceived value differences between officials and the public and between affluent and low-income groups. A separate cross-classification quantifies respondents who endorse all four ethical values and also classify bullying as mass/frequent.

At the beginning of 2025, Kazakhstan had 20.28 million residents. Ethnic Kazakhs comprised 71.3% of the population and ethnic Russians 14.6%; 12.77 million residents (63.0%) lived in urban localities and 7.51 million (37.0%) in rural localities Bureau of National Statistics of the Agency for Strategic Planning and Reforms of the Republic of Kazakhstan (2025d).

Average monthly per capita nominal income in 2024 was KZT 215,979, and the annual subsistence minimum was KZT 51,058. Consumption income fell below that threshold for 5.0% of the population. Regional per capita income varied by a factor of 3.3 in the fourth quarter of 2024 Bureau of National Statistics of the Agency for Strategic Planning and Reforms of the Republic of Kazakhstan (2025a), Bureau of National Statistics of the Agency for Strategic Planning and Reforms of the Republic of Kazakhstan (2025b), Bureau of National Statistics of the Agency for Strategic Planning and Reforms of the Republic of Kazakhstan (2025c). These figures document socioeconomic differentiation relevant to the affluent-poor measure.

Two items in the anti-value battery concern corruption and domestic violence. The Anti-Corruption Service registered 876 criminal offenses in the first half of 2024. Government data recorded nearly 100,000 domestic-violence reports and approximately 72,000 protective orders during 2024 (Anti-Corruption Agency of the Republic of Kazakhstan, 2024; Government of the Republic of Kazakhstan, 2024).

## 2 Theoretical framework

Classical bullying research uses schools, colleges, universities, and workplaces as its principal units of analysis. It examines perpetrator, victim, and bystander roles together with group norms, cohesion, and status structures (Hong and Espelage, 2012). Social-ecological models add meso-, exo-, and macro-level conditions, including inequality and cultural norms.

Research on school, classroom, and organizational climate analyzes shared beliefs about behavior that institutions and groups accept, encourage, or sanction. These beliefs are associated with aggression and bystander intervention alongside individual characteristics (Swearer and Hymel, 2015). At the societal level, the same analytical concept concerns shared perceptions of humiliation, violence, and abuse of power across institutions.

In this study, normative climate denotes the distribution of beliefs about how frequently humiliation, violence, and abuse of power occur. A widespread or frequent classification indicates perceived typicality; the item does not measure moral approval.

The term “moral economy of disrespect” denotes a configuration of status hierarchy, unequal access to protection, institutional distrust, and expectations of humiliating treatment (Sayer, 2007; Fassin, 2009; Karandinos et al., 2014; Palomera and Vetta, 2016). Cultural and symbolic violence and recognition theory explain how unequal status and disrespect acquire social intelligibility (Galtung, 1990; Bourdieu, 1991; Honneth, 1995, 2007).

Schemas learned in schools and workplaces may also organize relations between superiors and subordinates, officials and citizens, and affluent and low-income groups. Studies linking bullying to hierarchy and socioeconomic inequality provide an empirical basis for examining these relations (Due et al., 2009; Elgar et al., 2009, 2015; Hosozawa et al., 2021). Expectations that high-status or institutionally powerful actors can avoid sanctions may increase perceptions that humiliating conduct occurs routinely.

Kazakhstan's multiethnic and multicultural context includes official references to universal and national values, the state-forming role of Kazakh culture, and cultural diversity (Buribayev et al., 2025). Survey research records overlapping Kazakh, Russian, and other ethnocultural traditions in citizens' descriptions of everyday life (Adilova et al., 2021).

The study represents values through four declared priorities: responsibility; decency; honesty and selflessness; and social justice. Two additional items measure perceived value differences between officials and the public and between affluent and low-income groups. The anti-value battery includes “rejection of traditional values,” and its references to Kazakh, Russian, and other traditions concern overlapping ethnocultural repertoires within Kazakhstan.

Research on contemporary Kazakhstan reports family solidarity, cultural continuity, and intergenerational respect alongside education, self-reliance, career orientation, civic equality, and self-realization (Adilova et al., 2021; Buribayev et al., 2025). The analysis treats value continuity and change as coexisting dimensions of the national value structure.

A respondent met the ethical-core criterion after rating all four ethical items as “very important.” Cross-classification then counted respondents who met this criterion and classified bullying as mass/frequent. The analysis uses the term “ethical-core coexistence group” for this joint response pattern.

The binary mass/frequent bullying classification served as the outcome. Principal predictors were the anti-value prevalence cluster, the two perceived value-difference measures, and the ethical-core indicator; the model also included sociodemographic covariates and regional fixed effects.

H1 predicts higher odds of a mass/frequent bullying classification in the medium and high anti-value clusters relative to the low cluster. H2 predicts higher odds among respondents who perceive value differences between officials and the public or between affluent and low-income groups. The ethical-core cross-classification is descriptive and reports exact counts and proportions.

## 3 Methods

### 3.1 Study design and sampling

Data came from a cross-sectional, nationwide, quota-controlled survey conducted through standardized face-to-face computer-assisted personal interviews from May to June 2025. Eligible participants were Kazakhstan residents aged 18 years or older who had lived in the country for at least 6 months. The final sample included 3,123 respondents from all 17 regions and the cities of republican significance Astana, Almaty, and Shymkent. Gender, age, and ethnicity quotas followed official statistics. Logical consistency checks and data cleaning preceded analysis. Descriptive and inferential analyses used the unweighted sample.

### 3.2 Measures and covariates

Subjective household financial status recorded self-assessed purchasing capacity as subjectively affluent, middle, subjectively constrained, or refusal. Table 1 reports gender, education, ethnicity, settlement type, and financial status; ethnicity appears only in descriptive analyses.

**Table 1:** Sample characteristics and mass/frequent bullying classifications by respondent subgroup.

Characteristic/category	Full sample, *n* (%) *N* = 3,123	Primary sample, *n* (%) *N* = 2,798	Mass/frequent, *n* (row %)	Unadjusted OR (95% CI)
Age, years, mean (SD)	38.6 (13.8)	38.0 (13.8)	NA	NA
Ethnicity (descriptive)	
Kazakh	2,202 (70.5)	1,974 (70.6)	1,558 (78.9)	NA
Russian	463 (14.8)	417 (14.9)	320 (76.7)	NA
Other ethnicity	434 (13.9)	385 (13.8)	270 (70.1)	NA
Mixed/undetermined	24 (0.8)	22 (0.8)	15 (68.2)	NA
Gender	
Male	1,442 (46.2)	1,284 (45.9)	970 (75.5)	1.00 (reference)
Female	1,681 (53.8)	1,514 (54.1)	1,193 (78.8)	1.20 (1.01–1.44)
Education	
Secondary specialized or lower	1,536 (49.2)	1,340 (47.9)	1,020 (76.1)	1.00 (reference)
Higher/incomplete higher	1,587 (50.8)	1,458 (52.1)	1,143 (78.4)	1.14 (0.95–1.36)
Settlement type	
Metropolis	747 (23.9)	675 (24.1)	521 (77.2)	1.00 (reference)
Regional center	1,302 (41.7)	1,184 (42.3)	935 (79.0)	1.11 (0.88–1.39)
Other city/small town	1,074 (34.4)	939 (33.6)	707 (75.3)	0.90 (0.71–1.14)
Subjective household financial status	
Subjectively affluent	910 (29.1)	834 (29.8)	659 (79.0)	1.00 (reference)
Middle	1,626 (52.1)	1,476 (52.8)	1,160 (78.6)	0.97 (0.79–1.20)
Subjectively constrained	333 (10.7)	300 (10.7)	201 (67.0)	0.54 (0.40–0.72)
Refused	254 (8.1)	188 (6.7)	143 (76.1)	0.84 (0.58–1.23)

Percentages describe the unweighted quota-controlled sample. The primary sample excludes 325 difficult-to-answer bullying responses. Unadjusted odds ratios use isolated manifestations as the reference outcome. Ethnicity appears only in descriptive analyses. Global bivariate tests: gender chi-square (1) = 4.01, *p* = 0.045, Cramer's *V* = 0.038; education chi-square (1) = 1.93, *p* = 0.164, *V* = 0.026; settlement chi-square (2) = 4.04, *p* = 0.132, *V* = 0.038; financial status chi-square (3) = 21.11, *p* < 0.001, *V* = 0.087. Yates correction was applied to 2 × 2 tables. Supplementary Table S1 documents category construction.NA denotes not applicable.

Settlement type was derived from Q29 and grouped as metropolis, regional center, or other city/small town. The final category combined city and small-town responses and entered the model as a location covariate.

All analyses used the anonymized individual-level survey matrix. Khamzina et al. (2026) used the same matrix to examine normative priorities and perceived social cleavages.

Respondents completed the questionnaire in Russian or Kazakh. Supplementary Table S2 contains the Russian source wording, response options, and English analytical translations for every item analyzed here.

The bullying item offered four responses: “mass and ubiquitous; has become a stable trend,” “occurs frequently in various social groups,” “has isolated manifestations,” and “difficult to answer.” Descriptive analyses retained all four categories. The primary outcome coded the first two categories as Y = 1 and isolated manifestations as Y = 0. Exclusion of 325 difficult-to-answer responses produced an analytical sample of 2,798. A sensitivity model assigned difficult-to-answer responses to Y = 0 and retained all 3,123 participants. In this article, “perceived prevalence” refers to respondents' assessments of routine occurrence at the societal level.

The anti-value prevalence index counted mass/ubiquitous or frequent classifications across the nine non-bullying anti-values. Scores ranged from 0 to 9. Values of 0–2 formed the low cluster, 3–5 the medium cluster, and 6–9 the high cluster. Isolated and difficult-to-answer responses contributed zero points. The index excluded the bullying item.

The two perceived value-difference measures entered the model as categorical predictors. Response categories were large differences, minor differences, no differences, and difficult to answer; no differences served as the reference. Covariates included gender, age per decade, education, settlement type, subjective financial status, and fixed effects for 20 regional units. Reference categories were male, secondary specialized or lower education, metropolis, and subjectively affluent status. Supplementary Table S1 gives source codes and recoding rules.

The ethical-core indicator equaled 1 when a respondent rated responsibility, decency, honesty and selflessness, and social justice as “very important.” The indicator entered the regression as a predictor. Exact cross-classification with the four original bullying responses produced the ethical-core coexistence counts.

The analysis included one bullying outcome, nine anti-value indicators, two perceived value-difference measures, four ethical items, and six sociodemographic or location variables. KR-20/Cronbach's alpha for the nine binary anti-value indicators was 0.88 (*N* = 3,123). Cronbach's alpha for the four ethical items was 0.69 after difficult-to-answer responses were treated as missing (complete-case *n* = 3,075). Both composite variables entered the analysis according to the scoring rules stated above.

### 3.3 Statistical analysis

Binary logistic regression estimated adjusted associations with the mass/frequent bullying outcome. Predictors comprised the anti-value cluster, the two perceived value-difference measures, the ethical-core indicator, the specified covariates, and regional fixed effects. Maximum likelihood estimation used the primary analytical sample.


$$\begin{array}{l} \text{logit}\left[\text{P}\left(\text{Y\_i}=\text{1}\right)\right]=\text{ ​​}\beta \text{​​}0\;+\text{ ​}\beta \text{​​1Anti Value Cluster\_i}\\ \;+\text{​​}\beta \text{​​2 Officials Public\_i+ ​​}\beta \text{​​3Affluent Poor\_i}\\ \;+\beta \text{​​4 Ethical Core}\_\text{i+ ​​}\gamma \text{​​}\prime \text{X}\_\text{i + ​​}\delta \text{​}\_\text{r.} \end{array}$$

The anti-value cluster coefficients evaluated H1, and coefficients for the two perceived value-difference measures evaluated H2. Exact cross-classification described coexistence of the ethical-core criterion and the four bullying responses. The sensitivity model retained difficult-to-answer bullying responses in the reference outcome.

Bivariate associations were estimated with Pearson's chi-square test, Cramer's *V*, and unadjusted odds ratios with 95% confidence intervals. The logistic model reports adjusted odds ratios, 95% confidence intervals, and two-sided *p*-values using α = 0.05. Calculations were performed on unrounded respondent-level data in Python 3.13.5 (Python Software Foundation, Wilmington, DE, USA), using SciPy 1.17.0 (SciPy Developers, open-source community) and statsmodels 0.14.6 (statsmodels Development Team, open-source community). Subgroup analyses covered gender, education, settlement type, and subjective financial status through bivariate estimates, individual adjusted coefficients, and joint Wald tests.

## 4 Results

The sample included 53.8% female respondents and had a mean age of 38.6 years. The higher/incomplete higher education category represented 50.8%, including 42.4% with completed higher education. Respondents identified as Kazakh (70.5%), Russian (14.8%), another ethnicity (13.9%), or mixed/undetermined ethnicity (0.8%). Metropolises accounted for 23.9% of the sample, regional centers 41.7%, and other cities/small towns 34.4%. Subjective household financial status was affluent for 29.1%, middle for 52.1%, constrained for 10.7%, and refused by 8.1%. Table 1 gives primary-sample distributions and subgroup outcome estimates.

Among 3,123 respondents, 1,068 (34.2%) classified bullying as mass and ubiquitous, 1,095 (35.1%) as frequent, 635 (20.3%) as isolated, and 325 (10.4%) selected “difficult to answer.” The combined mass/frequent category comprised 2,163 respondents, or 69.3% of the full sample. In the primary analytical sample of 2,798 substantive responses, the corresponding proportion was 77.3%.

Table 2 reports the complete anti-value inventory. Mass/frequent classifications were most common for corruption (80.9%), indifference to pressing societal problems (74.9%), social dependency (72.8%), and hypocrisy and double standards (71.4%). Bullying ranked fifth at 69.3%.

**Table 2:** Perceived prevalence of bullying and other anti-values, exact *n* (%; *N* = 3,123).

Anti-value	Mass/ubiquitous	Frequent	Isolated	Difficult to answer	Mass/frequent
Corruption	1,753 (56.1)	773 (24.8)	410 (13.1)	187 (6.0)	2,526 (80.9)
Social dependency	1,062 (34.0)	1,212 (38.8)	525 (16.8)	324 (10.4)	2,274 (72.8)
Infantilism and social immaturity	936 (30.0)	1,162 (37.2)	622 (19.9)	403 (12.9)	2,098 (67.2)
Rejection of traditional values	797 (25.5)	1,001 (32.1)	1,034 (33.1)	291 (9.3)	1,798 (57.6)
Hypocrisy and double standards	1,152 (36.9)	1,077 (34.5)	635 (20.3)	259 (8.3)	2,229 (71.4)
Unwillingness to take responsibility for oneself and one's family	1,031 (33.0)	1,116 (35.7)	812 (26.0)	164 (5.3)	2,147 (68.7)
Indifference to pressing societal problems	1,192 (38.2)	1,148 (36.8)	605 (19.4)	178 (5.7)	2,340 (74.9)
Breakdown of traditional family formats and relations	945 (30.3)	1,118 (35.8)	852 (27.3)	208 (6.7)	2,063 (66.1)
Bullying	1,068 (34.2)	1,095 (35.1)	635 (20.3)	325 (10.4)	2,163 (69.3)
Domestic violence	1,009 (32.3)	1,091 (34.9)	796 (25.5)	227 (7.3)	2,100 (67.2)

“Mass/frequent” combines the mass/ubiquitous and frequent categories. Percentages use individual-level counts and are rounded to one decimal; row totals may equal 99.9 or 100.1%.

**Figure 1:**
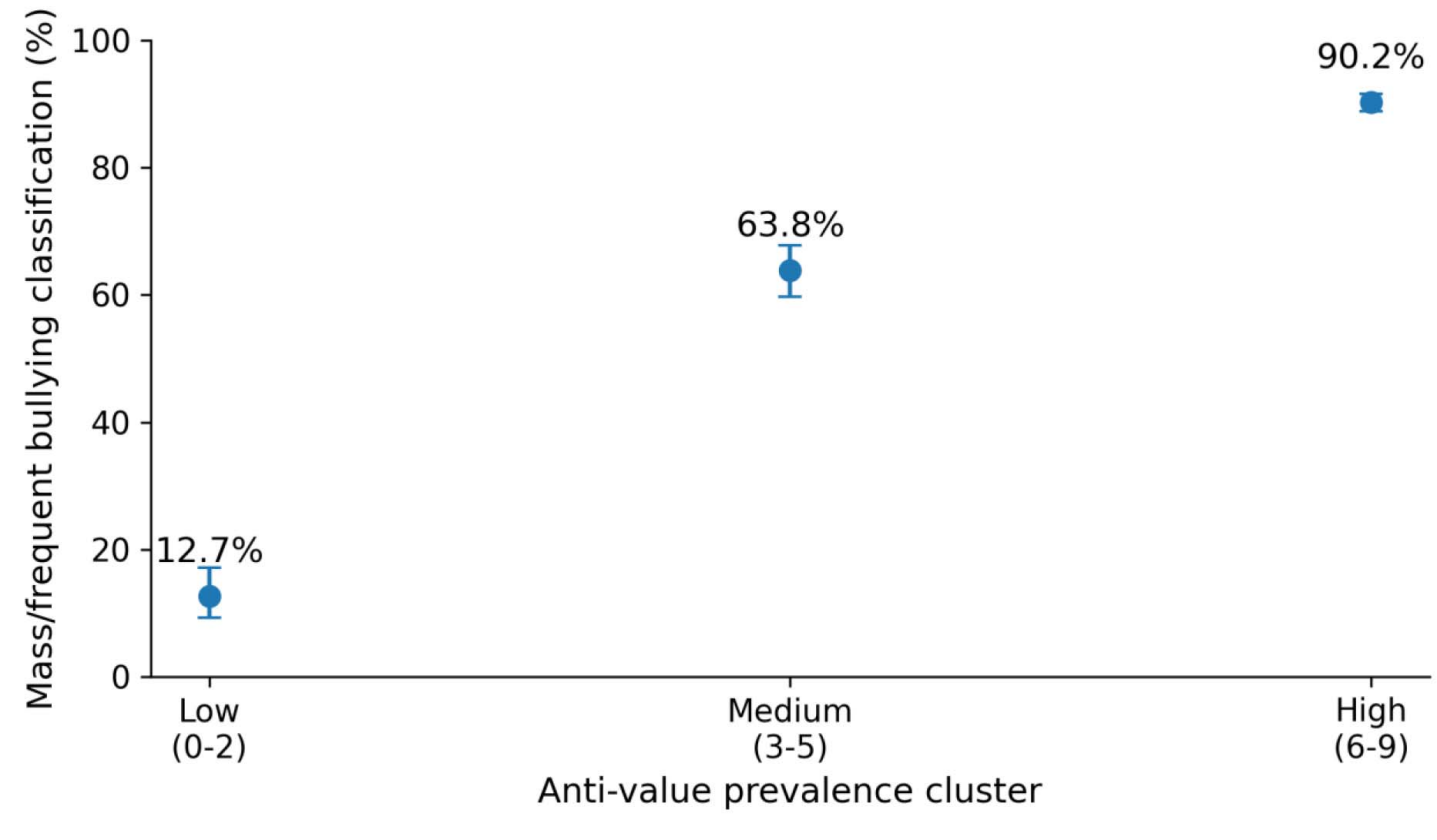
Observed mass/frequent bullying proportions by anti-value prevalence cluster in the primary analytical sample (*N* = 2,798). Points indicate observed proportions; whiskers indicate 95% Wilson binomial intervals: low, 12.7% (9.3–17.1); medium, 63.8% (59.7–67.8); high, 90.2% (88.8–91.5). The figure excludes difficult-to-answer bullying responses and omits bullying from the anti-value count.

Mass/frequent bullying classifications increased across the anti-value clusters: 12.7% in the low cluster, 63.8% in the medium cluster, and 90.2% in the high cluster (Figure 1). The association was chi-square (2, *N* = 2,798) = 916.91, *p* < 0.001, with Cramer's *V* = 0.572.

Using the low cluster as the reference, the unadjusted odds ratio was 12.10 for the medium cluster (95% CI 8.18–17.90) and 63.39 for the high cluster (95% CI 43.35–92.68). Table 3 provides exact cell counts (Figure 1).

**Table 3:** Mass/frequent bullying classification by anti-value prevalence cluster.

Anti-value prevalence cluster	*N*	Mass/frequent, *n* (row %)	Isolated, *n* (row %)	Unadjusted OR (95% CI)
Low (0–2)	283	36 (12.7)	247 (87.3)	1.00 (reference)
Medium (3–5)	539	344 (63.8)	195 (36.2)	12.10 (8.18–17.90)
High (6–9)	1,976	1,783 (90.2)	193 (9.8)	63.39 (43.35–92.68)
Total	2,798	2,163 (77.3)	635 (22.7)	

The primary outcome excludes difficult-to-answer responses. The anti-value index excludes bullying. Pearson chi-square (2, *N* = 2,798) = 916.91, *p* < 0.001; Cramer's *V* = 0.572. Odds ratios are unadjusted.

Mass/frequent classifications also varied across both perceived value-difference measures. For affluent-poor value differences, the proportions were 53.3% for no differences, 74.6% for minor differences, 79.9% for large differences, and 65.9% for difficult to answer; Pearson chi-square (3, *N* = 2,798) = 63.10, *p* < 0.001, Cramer's *V* = 0.150. For officials-public value differences, the corresponding proportions were 57.2%, 73.9%, 79.3%, and 64.4%; Pearson chi-square (3, *N* = 2,798) = 43.58, *p* < 0.001, Cramer's *V* = 0.125.

Relative to no differences, the unadjusted odds ratio for large differences was 3.47 (95% CI 2.48–4.86) for the affluent-poor measure and 2.87 (95% CI 2.02–4.08) for the officials-public measure. Table 4 reports all categories.

**Table 4:** Mass/frequent bullying classification by perceived value differences.

Cleavage and response category	*N*	Mass/frequent, *n* (row %)	Isolated, *n* (row %)	Unadjusted OR (95% CI)
Panel A. Affluent-poor value differences	
No differences	152	81 (53.3)	71 (46.7)	1.00 (reference)
Minor differences	468	349 (74.6)	119 (25.4)	2.57 (1.76–3.76)
Large differences	2,134	1,704 (79.9)	430 (20.1)	3.47 (2.48–4.86)
Difficult to answer	44	29 (65.9)	15 (34.1)	1.69 (0.84–3.41)
Panel B. Officials-public value differences	
No differences	138	79 (57.2)	59 (42.8)	1.00 (reference)
Minor differences	360	266 (73.9)	94 (26.1)	2.11 (1.40–3.19)
Large differences	2,255	1,789 (79.3)	466 (20.7)	2.87 (2.02–4.08)
Difficult to answer	45	29 (64.4)	16 (35.6)	1.35 (0.67–2.72)

Difficult-to-answer bullying responses are excluded. Panel A: Pearson chi-square (3, *N* = 2,798) = 63.10, *p* < 0.001, Cramer's *V* = 0.150. Panel B: Pearson chi-square (3, *N* = 2,798) = 43.58, *p* < 0.001, Cramer's *V* = 0.125. Odds ratios are unadjusted.

In the adjusted model (Table 5), the medium anti-value cluster had an aOR of 12.75 (95% CI 8.36–19.44), and the high cluster had an aOR of 64.07 (95% CI 42.50–96.58), each relative to the low cluster. Large affluent-poor value differences were associated with higher odds of a mass/frequent classification (aOR = 2.18, 95% CI 1.16–4.09, *p* = 0.016). Officials-public estimates were 0.88 for minor differences (95% CI 0.43–1.79, *p* = 0.721) and 0.77 for large differences (95% CI 0.39–1.53, *p* = 0.453). The ethical-core indicator had an aOR of 0.95 (95% CI 0.73–1.24, *p* = 0.720).

**Table 5:** Adjusted logistic regression for a mass/frequent bullying classification.

Predictor	aOR	95% CI	*p*-Value
Anti-value cluster: low (0–2)	1.00	Reference	
Anti-value cluster: medium (3–5)	12.75	8.36–19.44	< 0.001
Anti-value cluster: high (6–9)	64.07	42.50–96.58	< 0.001
Officials-public: no differences	1.00	Reference	
Officials-public: minor differences	0.88	0.43–1.79	0.721
Officials-public: large differences	0.77	0.39–1.53	0.453
Officials-public: difficult to answer	0.62	0.20–1.95	0.413
Affluent-poor: no differences	1.00	Reference	
Affluent-poor: minor differences	1.76	0.93–3.35	0.085
Affluent-poor: large differences	2.18	1.16–4.09	0.016
Affluent-poor: difficult to answer	2.29	0.79–6.66	0.127
Ethical core: yes (reference = no)	0.95	0.73–1.24	0.720
Female (reference = male)	1.30	1.03–1.62	0.024
Age (per 10 years)	0.99	0.91–1.07	0.826
Higher/incomplete higher education	1.11	0.88–1.41	0.377
Settlement: metropolis	1.00	Reference	
Settlement: regional center	2.61	1.28–5.35	0.009
Settlement: other city/small town	2.27	1.11–4.67	0.025
Subjective financial status: affluent	1.00	Reference	
Subjective financial status: middle	0.85	0.64–1.12	0.242
Subjective financial status: constrained	1.00	0.65–1.54	0.991
Subjective financial status: refused	1.27	0.76–2.11	0.366

*N* = 2,798; *Y* = 1 for mass/ubiquitous or frequent and *Y* = 0 for isolated manifestations. All estimates come from one model containing the displayed predictors and 20-region fixed effects. Likelihood-ratio chi-square (36) = 917.40, *p* < 0.001; McFadden pseudo-R-squared = 0.306; AIC = 2,153.61. Joint Wald tests: gender chi-square (1) = 5.06, *p* = 0.024; education chi-square (1) = 0.78, *p* = 0.377; settlement type chi-square (2) = 7.28, *p* = 0.026; subjective financial status chi-square (3) = 3.58,*p* = 0.311. Estimates are unweighted.

Female respondents had higher adjusted odds than male respondents (aOR = 1.30, 95% CI 1.03–1.62, *p* = 0.024). Relative to metropolises, regional centers had an aOR of 2.61 (95% CI 1.28–5.35, *p* = 0.009), and other cities/small towns had an aOR of 2.27 (95% CI 1.11–4.67, *p* = 0.025). Estimates for age, education, and subjective financial status were nonsignificant.

Joint Wald tests yielded *p* = 0.024 for gender and *p* = 0.026 for settlement type. The corresponding *p*-values were 0.377 for education and 0.311 for subjective financial status.

The sensitivity model produced aORs of 14.03 (95% CI 9.43–20.86) for the medium anti-value cluster and 68.07 (95% CI 46.27–100.14) for the high cluster. The estimate for large affluent-poor value differences was 1.67 (95% CI 0.95–2.92, *p* = 0.074). The estimate for large officials-public value differences was 1.06 (95% CI 0.59–1.91, *p* = 0.851).

Among 3,123 respondents, 2,300 (73.6%) met the ethical-core criterion. Of this group, 1,589 classified bullying as mass/frequent, representing 69.1% of ethical-core respondents and 50.9% of the full sample (Table 6). The association between ethical-core status and the four-category bullying response was nonsignificant, Pearson chi-square (3, *N* = 3,123) = 3.81, *p* = 0.283, Cramer's *V* = 0.035.

**Table 6:** Exact cross-classification of ethical-core status and bullying responses.

Ethical-core status	Mass/ubiquitous	Frequent	Isolated	Difficult to answer	Total
No	268 (32.6)	306 (37.2)	157 (19.1)	92 (11.2)	823
Yes	800 (34.8)	789 (34.3)	478 (20.8)	233 (10.1)	2,300
Total	1,068 (34.2)	1,095 (35.1)	635 (20.3)	325 (10.4)	3,123

Cells report n (row %). The ethical-core coexistence group includes 1,589 respondents in the mass/ubiquitous or frequent categories. Pearson chi-square (3, *N* = 3,123) = 3.81, *p* = 0.283; Cramer's *V* = 0.035.

## 5 Discussion

The principal association concerned the anti-value prevalence cluster. Relative to the low cluster, adjusted odds of a mass/frequent bullying classification were 12.75 in the medium cluster and 64.07 in the high cluster. Adjustment included perceived value differences, ethical-core status, sociodemographic covariates, and regional fixed effects. The estimates indicate that respondents' bullying assessments formed part of a concentrated response pattern across the nine other anti-values.

Large perceived value differences between affluent and low-income groups retained an adjusted association with the outcome (aOR = 2.18, 95% CI 1.16–4.09). Previous cross-national studies have linked bullying exposure to socioeconomic position, income inequality, and national wealth (Due et al., 2009; Elgar et al., 2009, 2015; Hosozawa et al., 2021). The officials-public measure showed bivariate associations; its adjusted coefficients were nonsignificant. In the sensitivity model, the affluent-poor estimate decreased to 1.67 and had *p* = 0.074.

Of the 2,300 respondents who met the ethical-core criterion, 1,589 (69.1%) also classified bullying as mass/frequent. Ethical-core status had no association with the four-category bullying response or with the adjusted binary outcome. The cross-classification documents frequent coexistence of declared ethical priorities and perceptions of widespread bullying across the sample.

Bullying and the nine anti-values were assessed in one battery with parallel response categories. This common measurement structure can contribute to the high cluster coefficients by capturing response consistency across socially disapproved practices. The survey did not measure personal exposure, media attention, response tendencies, or institutional trust, so the relative contribution of these mechanisms remains unknown.

Gender and settlement type retained adjusted associations. Female respondents had 1.30 times the odds observed among male respondents. Respondents in regional centers and other cities/small towns had more than twice the odds observed in metropolises. Age, education, and subjective household financial status yielded nonsignificant estimates. The simultaneous model separates the societal affluent-poor judgment from the respondent's household financial-status category.

### 5.1 Scope and limitations

The unit of inference is adults' cross-sectional assessment of societal prevalence. Personal victimization and observed incidence were not measured by the outcome. The design cannot establish temporal or causal order. The unweighted quota-controlled sample permits analysis of the observed associations, with caution required for population estimates. Parallel response formats for bullying and other anti-values may increase common-method covariance.

### 5.2 Policy and research implications

The association with perceived affluent-poor value differences makes access to protection and case handling relevant to school- and workplace-based prevention. Prevention programs require accessible reporting channels, documented investigation procedures, protection from retaliation, and consistent sanctions for abuse of power. Evaluations can measure reporting rates, processing time, case outcomes, and disparities by socioeconomic position. Longitudinal and intervention studies should use behavior-specific bullying measures, direct indicators of institutional trust and area-level inequality, bilingual cognitive testing, and comparative samples of adolescents and adults.

## 6 Conclusion

In the nationwide sample, 69.3% classified bullying as mass/frequent. Relative to the low anti-value cluster, adjusted odds were 12.75 in the medium cluster and 64.07 in the high cluster. Large perceived value differences between affluent and low-income groups were associated with higher odds of the same classification (aOR = 2.18, 95% CI 1.16–4.09). Adjusted officials-public estimates were nonsignificant.

Among respondents who endorsed all four ethical values as very important, 1,589 classified bullying as mass/frequent; this group represented 50.9% of the full sample and 69.1% of ethical-core respondents. The study identifies a concentrated pattern of perceived bullying and other anti-values, accompanied by a separate association with perceived affluent-poor value differences. Subsequent research should test these patterns with longitudinal data, behavior-specific measures, and direct indicators of institutional protection and socioeconomic inequality.

## Data Availability

The original contributions presented in the study are included in the article/Supplementary material, further inquiries can be directed to the corresponding author.
