# Supplementary material for: Perceived prevalence of bullying as a proxy for normalized disrespect: anti-values, social cleavages, and the normative climate of Kazakhstani society evidence from a nationwide survey
Source: Front Sociol. 2026 Aug 25;11:1872912. doi: 10.3389/fsoc.2026.1872912 (PMC13551876; doi:10.3389/fsoc.2026.1872912)
Supplement: Supplementary file 1 [file Table_1.docx]

**Supplementary Material**

Perceived Prevalence of Bullying as a Proxy for Normalized Disrespect: Anti-values, Social Cleavages, and the Normative Climate of Kazakhstani Society

Zhanna Khamzina and Yermek Buribayev

**Supplementary Table S1. Variable-level codebook and analytical recoding**

Source variable codes refer to the second header row of the supplied anonymized individual-level .xlsx matrix. The matrix contains 3,123 valid respondent records. The primary inferential sample contains 2,798 records after excluding the 325 respondents who selected “difficult to answer” for the bullying outcome; the sensitivity model retains all 3,123 respondents.

| **Construct / analytical role** | **Source variable(s)** | **Original survey item or category** | **Original coding** | **Analytical coding used in the manuscript** |
| --- | --- | --- | --- | --- |
| Perceived bullying normalization: primary and sensitivity outcomes | Q13.R9 | Bullying | 1 = mass/ubiquitous; 2 = frequent; 3 = isolated manifestations; 4 = difficult to answer. | Primary: Y = 1 for codes 1-2; Y = 0 for code 3; code 4 excluded (n = 2,798). Sensitivity: Y = 1 for codes 1-2; Y = 0 for codes 3-4 (n = 3,123). |
| Component of anti-value normalization index | Q13.R1 | Corruption | 1 = mass/ubiquitous; 2 = frequent; 3 = isolated manifestations; 4 = difficult to answer. | Item indicator = 1 for codes 1-2 and 0 for codes 3-4. Code 4 is treated as absence of an explicit normalization classification, not as a substantive judgment that the practice is rare. |
| Component of anti-value normalization index | Q13.R2 | Social dependency | 1 = mass/ubiquitous; 2 = frequent; 3 = isolated manifestations; 4 = difficult to answer. | Item indicator = 1 for codes 1-2 and 0 for codes 3-4. Code 4 is treated as absence of an explicit normalization classification, not as a substantive judgment that the practice is rare. |
| Component of anti-value normalization index | Q13.R3 | Infantilism and social immaturity | 1 = mass/ubiquitous; 2 = frequent; 3 = isolated manifestations; 4 = difficult to answer. | Item indicator = 1 for codes 1-2 and 0 for codes 3-4. Code 4 is treated as absence of an explicit normalization classification, not as a substantive judgment that the practice is rare. |
| Component of anti-value normalization index | Q13.R4 | Rejection of traditional values | 1 = mass/ubiquitous; 2 = frequent; 3 = isolated manifestations; 4 = difficult to answer. | Item indicator = 1 for codes 1-2 and 0 for codes 3-4. Code 4 is treated as absence of an explicit normalization classification, not as a substantive judgment that the practice is rare. |
| Component of anti-value normalization index | Q13.R5 | Hypocrisy and double standards | 1 = mass/ubiquitous; 2 = frequent; 3 = isolated manifestations; 4 = difficult to answer. | Item indicator = 1 for codes 1-2 and 0 for codes 3-4. Code 4 is treated as absence of an explicit normalization classification, not as a substantive judgment that the practice is rare. |
| Component of anti-value normalization index | Q13.R6 | Unwillingness to take responsibility for oneself and one’s family | 1 = mass/ubiquitous; 2 = frequent; 3 = isolated manifestations; 4 = difficult to answer. | Item indicator = 1 for codes 1-2 and 0 for codes 3-4. Code 4 is treated as absence of an explicit normalization classification, not as a substantive judgment that the practice is rare. |
| Component of anti-value normalization index | Q13.R7 | Indifference to pressing societal problems | 1 = mass/ubiquitous; 2 = frequent; 3 = isolated manifestations; 4 = difficult to answer. | Item indicator = 1 for codes 1-2 and 0 for codes 3-4. Code 4 is treated as absence of an explicit normalization classification, not as a substantive judgment that the practice is rare. |
| Component of anti-value normalization index | Q13.R8 | Breakdown of traditional family formats and relations | 1 = mass/ubiquitous; 2 = frequent; 3 = isolated manifestations; 4 = difficult to answer. | Item indicator = 1 for codes 1-2 and 0 for codes 3-4. Code 4 is treated as absence of an explicit normalization classification, not as a substantive judgment that the practice is rare. |
| Component of anti-value normalization index | Q13.R10 | Domestic violence | 1 = mass/ubiquitous; 2 = frequent; 3 = isolated manifestations; 4 = difficult to answer. | Item indicator = 1 for codes 1-2 and 0 for codes 3-4. Code 4 is treated as absence of an explicit normalization classification, not as a substantive judgment that the practice is rare. |
| Anti-value normalization count and cluster | Derived from Q13.R1-Q13.R8 and Q13.R10 | Count of the nine non-bullying anti-values explicitly classified as mass/ubiquitous or frequent. | Derived count ranges from 0 to 9; bullying (Q13.R9) is excluded. | Low = 0-2 (reference); medium = 3-5; high = 6-9. |
| Affluent-poor perceived value cleavage | Q5.R9 | Value differences between affluent and low-income groups | 1 = large differences; 2 = differences exist but are not substantial; 3 = no differences; 4 = difficult to answer. | Categorical predictor. Code 3 is the reference; separate contrasts for codes 2, 1, and 4. |
| Officials-public perceived value cleavage | Q5.R10 | Value differences between officials and the public | 1 = large differences; 2 = differences exist but are not substantial; 3 = no differences; 4 = difficult to answer. | Categorical predictor. Code 3 is the reference; separate contrasts for codes 2, 1, and 4. |
| Ethical-core component | Q9.R3 | Decency | 1 = very important; 2 = important to some extent; 3 = not important at all; 4 = difficult to answer. | Component indicator = 1 only for original code 1. |
| Ethical-core component | Q9.R4 | Responsibility | 1 = very important; 2 = important to some extent; 3 = not important at all; 4 = difficult to answer. | Component indicator = 1 only for original code 1. |
| Ethical-core component | Q9.R9 | Honesty and selflessness | 1 = very important; 2 = important to some extent; 3 = not important at all; 4 = difficult to answer. | Component indicator = 1 only for original code 1. |
| Ethical-core component | Q10.R1 | Social justice | 1 = very important; 2 = important to some extent; 3 = not important at all; 4 = difficult to answer. | Component indicator = 1 only for original code 1. |
| Ethical core | Derived from Q9.R3, Q9.R4, Q9.R9, and Q10.R1 | Simultaneous highest-category endorsement of all four ethical values. | Derived binary variable. | 1 when all four source variables equal 1; 0 otherwise. “No” is the regression reference. |
| Moral-dissonance response profile | Derived from ethical core and Q13.R9 | Coexistence of the ethical core with a mass/frequent bullying assessment. | Derived descriptive profile. | 1 when ethical core = 1 and Q13.R9 is code 1 or 2. Used for exact cross-classification, not as a regression predictor. |
| Gender | Q17 | Respondent sex | 1 = male; 2 = female. | Female dummy (code 2) versus male reference (code 1). |
| Age | Q18 | Completed age in years | Numeric age. | Entered continuously per 10-year increase (Q18 / 10). |
| Ethnicity (descriptive sample composition) | Q19 | Respondent ethnicity/nationality | 1 = Kazakh; 2 = Russian; 3 = other ethnicity; 4 = mixed origin / unable to determine ethnicity. | Reported descriptively in Table 1; not entered into the adjusted model and not used to infer homogeneous ethnic value systems. |
| Education | Q21 | Highest completed education | 1 = academic degree; 2 = master’s; 3 = bachelor/specialist; 4 = incomplete higher; 5 = secondary specialized/vocational; 6 = general secondary; 7 = incomplete secondary; 8 = primary; 9 = no education. | Completed higher = codes 1-3 (n = 1,324; 42.4% of the full sample). Higher/incomplete higher = codes 1-4 (n = 1,587; 50.8%) for subgroup tests and the adjusted model; secondary specialized or lower = codes 5-9 (reference). |
| Settlement type | Q29 | Type of settlement | 1 = metropolis; 2 = regional center; 3 = city; 4 = small town; 5 = settlement/village. | Metropolis = reference; regional center = code 2; other city/small town = observed codes 3-4 combined. Code 5 was defined in the value-label dictionary but had 0 valid observations. Free-text locality names suggest that some apparently rural localities may have been coded as code 4; no authoritative recoding or rural-urban comparison was performed, and Q29 was not used to verify a settlement-type quota. |
| Subjective financial status | Q27 | Household financial status | 1 = no financial difficulties / can buy housing; 2 = can afford all except housing; 3 = can buy basic appliances but not a car; 4 = food and clothing affordable but major appliances difficult; 5 = food affordable but clothing difficult; 6 = sometimes insufficient even for food; 7 = refusal. | Subjectively affluent = codes 1-2 (reference); middle = codes 3-4; subjectively constrained = codes 5-6; refused = code 7. These are self-assessed household purchasing-capacity categories, not objective income classes or official poverty categories. |
| Regional fixed effects | Q28 | Administrative-territorial unit of residence | Twenty original region codes; see Panel B. | Entered as a 20-level categorical factor. Astana (code 1) is the treatment reference; the remaining 19 coefficients are included but not displayed in the main regression table. |
| Interview language | Not retained as a separate respondent-level field in the supplied analytical matrix | Fieldwork documentation states that interviews were administered in Russian or Kazakh according to respondent preference. | No respondent-level interview-language code was available in the analytical matrix. | Not entered into the regression. No language-stratified model or formal measurement-invariance test was estimated. |
| Weighting and analytical sample | No weight variable used | Nationwide sample with quota control documented for gender, age, and ethnicity | The supplied matrix contains 3,123 valid records. | All reported estimates are unweighted. Primary model n = 2,798; sensitivity model n = 3,123. No separate settlement-type quota variable was available, and Q29 does not verify a settlement-type quota. |

**Panel B. Q28 regional code crosswalk**

| **Code** | **Administrative unit** | **Code** | **Administrative unit** |
| --- | --- | --- | --- |
| 1 | Astana | 11 | Zhetysu Region |
| 2 | Almaty | 12 | Karaganda Region |
| 3 | Shymkent | 13 | Kostanay Region |
| 4 | Abai Region | 14 | Kyzylorda Region |
| 5 | Akmola Region | 15 | Mangystau Region |
| 6 | Aktobe Region | 16 | Pavlodar Region |
| 7 | Almaty Region | 17 | North Kazakhstan Region |
| 8 | Atyrau Region | 18 | Turkistan Region |
| 9 | West Kazakhstan Region | 19 | Ulytau Region |
| 10 | Zhambyl Region | 20 | East Kazakhstan Region |

**Panel C. Internal-consistency diagnostics**

| **Component** | **Items and scoring** | **N** | **Coefficient and interpretation** |
| --- | --- | --- | --- |
| **Anti-value normalization set** | Q13.R1-Q13.R8 and Q13.R10; codes 1-2 = 1 and codes 3-4 = 0. | 3,123 | KR-20/Cronbach’s alpha = .88. Descriptive reliability diagnostic for a formative count; not evidence of a reflective latent scale. |
| **Ethical-core source items** | Q9.R3, Q9.R4, Q9.R9, and Q10.R1 on their original four-category response scales; “difficult to answer” treated as missing. | 3,075 | Cronbach’s alpha = .69. Descriptive diagnostic only because the final ethical-core indicator is a conjunctive all-four-items rule. |
| **Single-item constructs** | Q13.R9, Q5.R9, and Q5.R10. | — | Internal-consistency alpha is not applicable to single-item measures. |

Notes. The codebook documents the 16 substantive source items used for the focal constructs, six sociodemographic/location fields entered in or defining the adjusted model, and one descriptive ethnicity field. It covers the variables used in Tables 1-6 and in the primary and sensitivity logistic models. Raw response labels are translated into English for reporting. Completed higher education (Q21 codes 1-3) comprised 1,324 respondents (42.4%); the subgroup tests and regression used the broader higher/incomplete higher category (codes 1-4), n = 1,587 (50.8%). The Q29 value-label dictionary defined code 5 (“settlement/village”), but no valid respondent had that code; free-text locality names suggest that some apparently rural localities may have been coded as “small town,” so the codebook does not treat Q29 as a reliable rural-urban classification. No separate settlement-type quota variable was available, and the supplement does not claim that such a quota was reliably implemented. The financial-status labels are subjective household assessments, not objective income classes or official poverty categories. The fieldwork report documents translation of the approved questionnaire into Kazakh by the contractor and agreement with the commissioning team, but the exact complete Kazakh-language field instrument is not confirmed by the materials available for this revision and has not been reconstructed; the documentation does not record formal back-translation, cognitive interviewing, or measurement-invariance testing. The reported reliability coefficients are descriptive diagnostics and do not change the formative/conjunctive construction of the two composite variables.

**Supplementary Table S2. Study-specific questionnaire items, source wording, response formats, and English analytical translations**

This table reproduces the complete source wording and response options for every item used in the present analysis. It is a study-specific item set, not the entire omnibus survey. The full Russian omnibus questionnaire is not reproduced in this supplement. Interviews were conducted in Russian or Kazakh according to respondent preference; the exact complete Kazakh-language field instrument was not included or otherwise confirmed in the materials available for this revision and has not been reconstructed. The English wording is an analytical translation and is not presented as a separately validated instrument.

| **Variable(s)** | **Russian source wording / item** | **English analytical translation** | **Response format / analytical role** |
| --- | --- | --- | --- |
| **Panel A. Perceived social cleavages** | | | |
| **Q5.R9** | Как вы думаете, какие различия есть между разными группами казахстанцев? (Отмечайте по каждой строке таблицы только 1 ответ). Item: Между обеспеченными и малоимущими слоями населения. | In your opinion, what differences exist between different groups of Kazakhstanis? (Select one response for each row.) Item: Between affluent and low-income population groups. | 1 = Различия большие; 2 = Различия имеются, но не существенные; 3 = Различий нет; 4 = Затрудняюсь ответить. Categorical predictors; code 3 is the reference. |
| **Q5.R10** | Как вы думаете, какие различия есть между разными группами казахстанцев? (Отмечайте по каждой строке таблицы только 1 ответ). Item: Между чиновниками и народом. | In your opinion, what differences exist between different groups of Kazakhstanis? (Select one response for each row.) Item: Between officials and the public. | 1 = Различия большие; 2 = Различия имеются, но не существенные; 3 = Различий нет; 4 = Затрудняюсь ответить. Categorical predictors; code 3 is the reference. |
| **Panel B. Ethical-core items** | | | |
| **Q9.R3** | Что и в какой степени стало важным для Вас и Вашей семьи в годы суверенного развития? (Один ответ по каждой строке). Item: Порядочность. | What, and to what extent, has become important for you and your family during the years of sovereign development? (One response for each row.) Item: Decency. | 1 = Очень важно; 2 = Важно отчасти; 3 = Совсем не важно; 4 = Затрудняюсь ответить. Ethical-core component equals 1 only for code 1. |
| **Q9.R4** | Что и в какой степени стало важным для Вас и Вашей семьи в годы суверенного развития? (Один ответ по каждой строке). Item: Ответственность. | What, and to what extent, has become important for you and your family during the years of sovereign development? (One response for each row.) Item: Responsibility. | 1 = Очень важно; 2 = Важно отчасти; 3 = Совсем не важно; 4 = Затрудняюсь ответить. Ethical-core component equals 1 only for code 1. |
| **Q9.R9** | Что и в какой степени стало важным для Вас и Вашей семьи в годы суверенного развития? (Один ответ по каждой строке). Item: Честность и бескорыстие. | What, and to what extent, has become important for you and your family during the years of sovereign development? (One response for each row.) Item: Honesty and selflessness. | 1 = Очень важно; 2 = Важно отчасти; 3 = Совсем не важно; 4 = Затрудняюсь ответить. Ethical-core component equals 1 only for code 1. |
| **Q10.R1** | Что и в какой степени стало важным для Вас и Вашей семьи в годы суверенного развития? (Один ответ по каждой строке). Item: Социальная справедливость. | What, and to what extent, has become important for you and your family during the years of sovereign development? (One response for each row.) Item: Social justice. | 1 = Очень важно; 2 = Важно отчасти; 3 = Совсем не важно; 4 = Затрудняюсь ответить. Ethical-core component equals 1 only for code 1. |
| **Panel C. Perceived prevalence of anti-values** | | | |
| **Q13.R1-R10** | Какие анти-ценности (установки, модели поведения) и в какой мере распространены на современном этапе развития казахстанского общества? (Отмечайте одно значение по каждой строке). | Which anti-values (attitudes and behavioral models), and to what extent, are widespread at the present stage of development of Kazakhstani society? (Select one response for each row.) | 1 = Имеет массовый повсеместный характер, стало устойчивой тенденцией; 2 = Встречается часто в разных социальных группах; 3 = Имеет единичные проявления; 4 = Затрудняюсь ответить. |
| **Q13.R1** | Коррупция. | Corruption. | Anti-value index component. |
| **Q13.R2** | Социальное иждивенчество. | Social dependency. | Anti-value index component. |
| **Q13.R3** | Инфантильность, социальная незрелость. | Infantilism and social immaturity. | Anti-value index component. |
| **Q13.R4** | Отказ от традиционных ценностей. | Rejection of traditional values. | Anti-value index component. |
| **Q13.R5** | Лицемерие и двойные стандарты в оценке событий в общественной и личной жизни. | Hypocrisy and double standards in evaluating events in public and personal life. | Anti-value index component. |
| **Q13.R6** | Нежелание брать ответственность за себя, за семью. | Unwillingness to take responsibility for oneself and one’s family. | Anti-value index component. |
| **Q13.R7** | Равнодушное отношение к актуальным проблемам общества. | Indifference to pressing societal problems. | Anti-value index component. |
| **Q13.R8** | Распад традиционных форматов семьи и семейных отношений. | Breakdown of traditional family formats and relations. | Anti-value index component. |
| **Q13.R9** | Буллинг. | Bullying. | Primary and sensitivity outcome. |
| **Q13.R10** | Насилие в семье. | Domestic violence. | Anti-value index component. |
| **Panel D. Sociodemographic and location variables** | | | |
| **Q17** | Пол. | Respondent sex. | 1 = Мужской; 2 = Женский. Female versus male reference. |
| **Q18** | Возраст. Сколько Вам полных лет? | Age. How old are you in completed years? | Numeric completed age; entered per 10-year increase. |
| **Q19** | Ваша национальность. | Your ethnicity/nationality. | 1 = Казах/казашка; 2 = Русский/русская; 3 = Другая национальность; 4 = Смешанное происхождение, не могу определить свою национальность. Descriptive composition only. |
| **Q21** | Ваше образование. | Your highest completed education. | 1 = Ученая степень (кандидат наук, доктор наук, PhD); 2 = Высшее со степенью магистра; 3 = Высшее со степенью бакалавра и специалиста; 4 = Незаконченное высшее; 5 = Среднее специальное, профессиональное образование; 6 = Общее среднее; 7 = Неполное среднее; 8 = Начальное; 9 = Нет образования. Codes 1-3 denote completed higher education (n = 1,324; 42.4%); analyses use codes 1-4 as higher/incomplete higher (n = 1,587; 50.8%) versus codes 5-9. |
| **Q27** | Как бы Вы оценили финансовый статус Вашей семьи? | How would you assess the financial status of your family? | 1 = У нас нет никаких финансовых затруднений. При необходимости мы можем купить квартиру или дом; 2 = Наших средств хватит на все, кроме таких дорогих приобретений, как квартира или загородный дом; 3 = Мы можем купить основную бытовую технику, но на автомобиль нам не хватит; 4 = Нам хватает на еду и одежду, но купить телевизор, холодильник или стиральную машину нам будет сложно; 5 = У нас хватает денег на еду, но купить одежду для нас - серьезная проблема; 6 = Нам не всегда достаточно денег даже на еду; 7 = Отказ от ответа. Recoded as subjectively affluent 1-2, middle 3-4, subjectively constrained 5-6, and refused 7. These are self-assessed household categories, not objective income classes or official poverty categories. |
| **Q28** | В каком регионе Вы проживаете? | In which region do you live? | Codes 1-20; administrative-unit crosswalk is provided in Supplementary Table S1, Panel B. Regional fixed effects. |
| **Q29** | Тип населённого пункта. | Type of settlement. | 1 = Мегаполис (население более 1 миллиона человек); 2 = Областной центр; 3 = Город; 4 = Малый город; 5 = Посёлок / Село. Model: metropolis reference; regional center; other city/small town (observed codes 3-4). Code 5 was defined but had 0 valid observations. Free-text locality names suggest that some apparently rural localities may have been coded as code 4; no authoritative recoding or rural-urban comparison was performed. |

*Note. The table reproduces every source item used in the reported analyses and is not the full omnibus questionnaire. Russian wording and response labels are preserved from the available analytical materials; punctuation and capitalization have been normalized only minimally for presentation. The full Russian omnibus questionnaire is not reproduced in this supplement. The exact complete Kazakh-language field instrument is not confirmed by the materials available for this revision and has not been reconstructed. English wording is an analytical translation.*
